# Association between creatinine-to-body weight ratio and incident prediabetes in Chinese adults: a large-scale retrospective cohort study

**DOI:** 10.3389/fendo.2026.1713333

**Published:** 2026-04-02

**Authors:** Wushan Pan, Yuheng Liao, Yong Han, Changchun Cao, Haofei Hu

**Affiliations:** 1 Department of Nephrology, Kaifeng Central Hospital, Kaifeng, Henan, China; 2 Department of Nephrology, Shenzhen Second People’s Hospital, Shenzhen, Guangdong, China; 3 Department of Nephrology, The First Affiliated Hospital of Shenzhen University, Shenzhen, Guangdong, China; 4 Department of Emergency, Shenzhen Second People’s Hospital, Shenzhen, Guangdong, China; 5 Department of Emergency, The First Affiliated Hospital of Shenzhen University, Shenzhen, Guangdong, China; 6 Department of Rehabilitation, The First Affiliated Hospital of Shenzhen University, Shenzhen, Guangdong, China; 7 Department of Rehabilitation, Shenzhen Second People’s Hospital, Shenzhen, Guangdong, China

**Keywords:** cohort study, creatinine-to-body weight ratio, muscle mass, non-linear relationship, prediabetes

## Abstract

**Background and objective:**

While the creatinine-to-body weight ratio (Cre/BW) has emerged as a promising biomarker for muscle mass assessment, its relationship with prediabetes remains unclear. This study aimed to investigate the association between Cre/BW ratio and incident prediabetes in Chinese adults.

**Methods:**

We conducted a large-scale retrospective cohort study involving 173,476 participants from health check-up programs across 11 Chinese cities. Cox proportional hazards models were employed to evaluate the association between baseline Cre/BW ratio and incident prediabetes. To address potential non-linear relationships, we applied Cox proportional hazards regression with cubic spline functions and smooth curve fitting, using a recursive algorithm to calculate inflection points. Multiple imputation was used for missing data, and comprehensive sensitivity analyses were performed to assess result robustness.

**Results:**

During a median follow-up of 3.0 years, 18,506 participants (10.67%) developed prediabetes. A lower Cre/BW ratio was associated with an increased risk of prediabetes (adjusted HR = 0.869, 95%CI: 0.806-0.973). Exploratory threshold effect analysis suggested a potential inflection point at 0.96(95% CI 0.90-1.01)μmol/L/kg, below which the association might be stronger (HR = 0.407, 95%CI: 0.328-0.506). The association remained stable in sensitivity analyses excluding participants with smoking history, drinking history, or family history of diabetes. Subgroup analyses revealed more pronounced associations among individuals aged 30–40 years (HR = 0.614, 95%CI: 0.532-0.708), females (HR = 0.726, 95%CI: 0.640-0.824), and those with normal blood pressure (systolic blood pressure <140 mmHg, HR = 0.816, 95%CI: 0.752-0.886).

**Conclusion:**

A lower Cre/BW ratio is associated with an increased risk of prediabetes in Chinese adults. Exploratory threshold effect analysis suggested a potential inflection point at 0.96(95% CI 0.90-1.01) μmol/L/kg. These findings suggest that the Cre/BW ratio could serve as a simple, cost-effective tool for prediabetes risk stratification in clinical practice.

## Background

Diabetes mellitus (DM) has become a major global public health challenge, and its prevalence has increased sharply over recent decades (1, 2). The International Diabetes Federation (IDF) estimated that, among adults aged 20–79 years, diabetes affected 10.5% of the population (537 million people) in 2021 and is projected to rise to 12.2% (783 million) by 2045 (3). In China, the world’s most populous nation, the diabetes burden is particularly severe, with recent national surveys indicating a prevalence of 11.2% for diabetes and an alarming 35.7% for prediabetes among adults (4).

Prediabetes, characterized by impaired fasting glucose (IFG) and/or impaired glucose tolerance (IGT), represents a critical transitional state between normal glucose metabolism and diabetes according to the American Diabetes Association (ADA) criteria (5, 6). Individuals with prediabetes face a 5-10% annual risk of progressing to type 2 diabetes mellitus (T2DM) (7). Therefore, early identification of high-risk individuals with prediabetes and implementation of effective interventions are crucial for diabetes prevention.

Recent research has increasingly focused on the link between muscle mass and metabolic disturbances (8, 9). Skeletal muscle, the body’s largest insulin-responsive tissue, is central to glucose homeostasis (10). Accumulating evidence indicates that reduced muscle mass is associated with higher risks of insulin resistance, impaired glucose tolerance, and T2DM (11, 12). However, traditional methods of assessing muscle mass, such as dual-energy X-ray absorptiometry (DXA) and bioelectrical impedance analysis (BIA), are limited in their application to large-scale population studies due to cost and logistical constraints.

The creatinine-to-body weight ratio (Cre/BW) has recently emerged as a simple and cost-effective surrogate marker for muscle mass (13). Serum creatinine, primarily produced by skeletal muscle, correlates positively with muscle mass (14). The Cre/BW ratio not only reflects muscle mass but also considers the weight factor, potentially offering a more accurate representation of the metabolic role of skeletal muscle (15).

Prior studies have examined associations between the Cre/BW ratio and a range of metabolic conditions. Hashimoto et al. reported that the Cre/BW ratio was inversely related to incident T2DM (13). In addition, another study showed that a lower Cre/BW ratio was linked to a higher risk of non-alcoholic fatty liver disease (16). However, research on the relationship between the Cre/BW ratio and prediabetes remains limited, particularly in large-scale studies among the general Chinese population. Recent longitudinal studies and population-based cohorts over the past few years have increasingly validated the utility of the Cre/BW ratio as a reliable, cost-effective surrogate for skeletal muscle mass in predicting cardiometabolic outcomes, including incident type 2 diabetes and insulin resistance (13, 17).

We hypothesized that a lower Cre/BW ratio might be inversely associated with the risk of prediabetes. This study aimed to investigate the relationship between this potential muscle mass surrogate and prediabetes susceptibility, and to explore its utility as a simple marker for risk stratification. These findings might contribute to future strategies for prediabetes screening and prevention in China and beyond.

## Methods

### Study design

This study was designed as a retrospective cohort analysis to evaluate the association between the baseline Cre/BW ratio and the risk of developing prediabetes over follow-up. The baseline Cre/BW ratio was treated as the exposure, and incident prediabetes during follow-up was defined as the outcome (time-to-event: 0 = no prediabetes, 1 = prediabetes).

### Data source

The study drew on health examination data from the Rich Healthcare Group database, which is publicly available via the DATADRYAD repository (https://datadryad.org/stash/dataset/doi:10.5061/dryad.ft8750v). This database encompasses standardized health examination information collected between 2010 and 2016. The Dryad repository provides non-commercial access to this dataset for academic researchers and permits its adaptation and the creation of derivative works, provided that the original source and authors are properly acknowledged (18).

### Study population

This multi-center study enrolled consecutive participants from 32 sites across 11 Chinese cities (Beijing, Changzhou, Chengdu, Guangzhou, Nanjing, Hefei, Nantong, Shenzhen, Shanghai, Suzhou, and Wuhan). The study data, derived from the Rich Healthcare Group’s electronic medical records database (2010–2016), was anonymized using non-traceable identification codes. The research protocol adhered to the Declaration of Helsinki guidelines and received approval from the Rich Healthcare Group’s clinical research ethics committee. Considering the retrospective study design and the use of de-identified data, the Institutional Review Board waived the need to obtain informed consent (18, 19).

**Figure 1:**
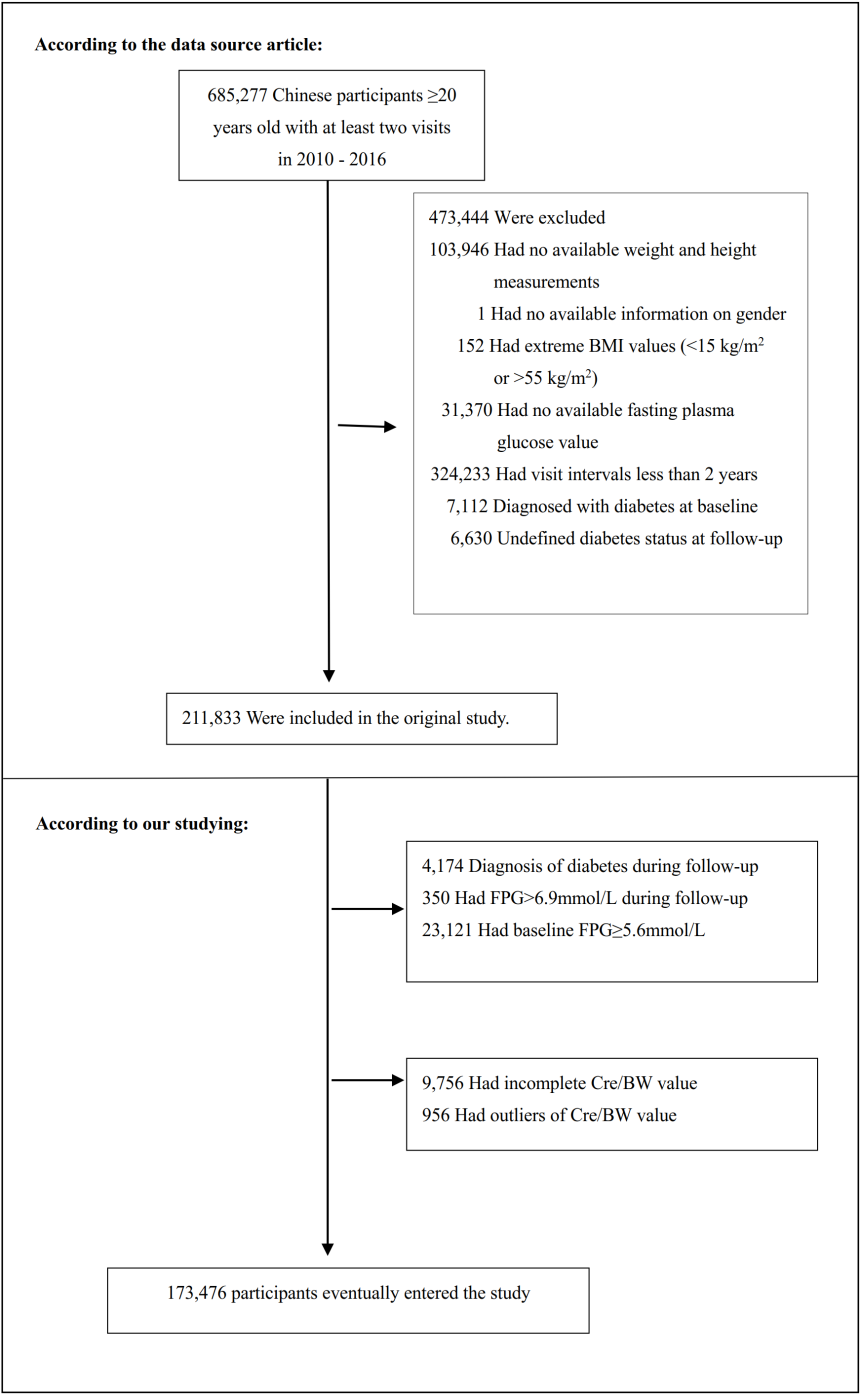
Flowchart of study participants. The systematic participant selection process is illustrated in Figure 1. The initial cohort consisted of 685,277 individuals who underwent at least two health examinations. After applying multiple predefined exclusion criteria, a final cohort of 173,476 eligible participants was established for subsequent analyses.

From an initial cohort of 685,277 participants who underwent multiple health examinations, we implemented a stepwise exclusion procedure. Participants were excluded according to the following criteria: (1) missing or indeterminate data on demographics, anthropometrics, laboratory parameters (fasting plasma glucose (FPG), Cre/BW ratio), or outcome status; (2) implausible or extreme values (e.g., extreme body mass index (BMI) or Cre/BW ratio outliers); (3) insufficient follow-up duration (<2 years); and (4) presence of diabetes or prediabetes at baseline (FPG ≥5.6 mmol/L), or progression to diabetes during follow-up. The final analytical cohort consisted of 173,476 participants. A detailed participant selection process and specific exclusion counts are presented in **Figure 1**.We have reinforced the justification for the exclusion criteria to clarify it is strictly for defining the incident cohort.

### Variables

#### Assessment of creatinine-to-body weight ratio and outcome measures

The Cre/BW ratio was calculated as serum creatinine (μmol/L) divided by body weight (kg) and analyzed as a continuous variable at baseline. Analyses were performed for Cre/BW as a continuous variable (per 1-μmol/L/kg increment) and as a categorical variable (quartiles).”. The primary endpoint was incident prediabetes, defined according to the American Diabetes Association’s 2018 criteria as impaired fasting glucose with FPG values ranging from 5.6 to 6.9 mmol/L (20). Participants were monitored for up to 5 years, with follow-up concluding at prediabetes diagnosis, final clinical visit, or December 31, 2016, whichever occurred first.

### Covariates

Covariate selection was informed by prior research and clinical expertise (13, 17, 21). The analyzed covariates comprised two categories: (1) Continuous variables: age, anthropometric measures (height), blood pressure parameters (systolic blood pressure (SBP) and diastolic blood pressure (DBP)), metabolic indices (FPG, blood urea nitrogen (BUN)), lipid profile (total cholesterol (TC), triglyceride(TG), high-density lipoprotein cholesterol (HDL-c), low-density lipoprotein cholesterol(LDL-c), and liver function markers (alanine aminotransferase(ALT), aspartate aminotransferase(AST)). (2) Categorical variables: demographic factors (gender), lifestyle characteristics (smoking and drinking status), and hereditary factors (family history of diabetes).

### Data collection and measurements

Trained healthcare personnel performed thorough baseline evaluations in accordance with standardized procedures. Anthropometric measurements were obtained with participants in light clothing without footwear. Height was measured to 0.1 cm precision using a stadiometer, while weight was recorded to 0.1 kg using calibrated electronic scales. Body mass index was computed as weight(kg)/height(m²). Blood pressure measurements were performed using standard mercury sphygmomanometers after participants maintained a seated position for 5 minutes.

Lifestyle factors were assessed through structured questionnaires at baseline. Smoking and alcohol consumption status were each categorized into three groups: current, former, or never users. Demographic information and medical history were similarly documented through standardized questionnaires.

Biochemical analyses were conducted on overnight fasting (≥10 hours) blood samples using a Beckman 5800 autoanalyzer (18). The assessed parameters included HDL-c, BUN, TC, AST, TG, Scr, FPG, LDL-c, and ALT.

### Missing data processing

The dataset exhibited different levels of missingness across variables. Missing data were minimal for physiological parameters: blood pressure measurements (n=15, <0.01%), total cholesterol (n=2,680, 1.5%), triglycerides (n=2,685, 1.6%), alanine aminotransferase (n=976, 0.6%), and blood urea nitrogen (n=9,504, 5.5%). More substantial missing data were observed for lipid parameters (HDL-c: n=74,455, 42.9%; LDL-c: n=73,970, 42.6%), liver function (AST: n=100,494, 57.9%), and lifestyle factors (smoking and drinking status: both n=124,935, 72.0%). To minimize potential bias and improve data efficiency, we applied multiple imputation by chained equations (MICE) (22). The imputation model incorporated age, sex, height, SBP, DBP, FPG, TC, TG, HDL-c, LDL-c, ALT, AST, BUN, smoking status, drinking status, and family history of diabetes. Missingness was evaluated under the assumption of missing at random (MAR) (23). Although the MAR assumption is strictly unverifiable from the observed data, we included all analysis variables and relevant auxiliary predictors in the imputation model to maximize the plausibility of this assumption.

### Statistical analysis

Participants were categorized into quartiles according to the Cre/BW ratio. In descriptive statistics, continuous variables with a normal distribution were reported as mean ± standard deviation, whereas non-normally distributed variables were described using the median (IQR). Categorical data were presented as counts and proportions. Comparisons among Cre/BW quartile groups used one-way ANOVA for normally distributed variables, the Kruskal–Wallis H test for skewed variables, and the χ² test for categorical variables. For time-to-event outcomes, Kaplan–Meier methods were applied, and prediabetes-free survival across Cre/BW quartiles was assessed with the log-rank test.

We assessed potential collinearity among covariates using variance inflation factors (VIF) (24), computed as 1/(1−R^2^), where R^2^was obtained from a series of linear regression models in which each variable was alternately treated as the outcome and regressed on all remaining variables. Variables with VIF >5 were removed from the multivariable regression analyses to mitigate collinearity (**Supplementary Table 1**).

The association between Cre/BW ratio and incident prediabetes was evaluated using Cox proportional hazards models, with results expressed as hazard ratios (HRs) and 95% confidence intervals (CIs). We implemented three sequential models to assess the association: Model I (unadjusted), Model II (adjusted for demographic and clinical factors), and Model III (fully adjusted for all potential confounders). The specific covariates adjusted in each model are detailed in the footnotes of **Table 1**. Covariate selection was guided by previous literature (13, 17, 21), and collinearity assessment. Total cholesterol was excluded from the multivariate analysis due to demonstrated collinearity (**Supplementary Table 1**). The proportional hazards assumptions were validated using Schoenfeld residuals and log-minus-log plots.

**Table 1:** Relationship between Cre/BW ratio and the incident prediabetes in different models.

Exposure	Model I(HR,95%CI, P)	Model II(HR,95%CI, P)	Model III(HR,95%CI, P)
Cre/BW ratio (per 1 μmol/L/kg increase)	0.721 (0.673, 0.772) <0.0001	0.602 (0.559, 0.648) <0.0001	0.869 (0.806, 0.937) 0.0003
Cre/BW ratio quartile			
Q1	Ref.	Ref.	Ref.
Q2	0.825 (0.793, 0.858) <0.0001	0.823 (0.791, 0.857) <0.0001	0.892 (0.857, 0.929) <0.0001
Q3	0.827 (0.795, 0.860) <0.0001	0.802 (0.770, 0.836) <0.0001	0.911 (0.874, 0.950) <0.0001
Q4	0.822 (0.789, 0.856) <0.0001	0.752 (0.720, 0.785) <0.0001	0.924 (0.884, 0.966) 0.0005
P for trend	<0.0001	<0.0001	0.0010

Model I: we did not adjust other covariates.

Model II: we adjust age, gender, height, SBP, DBP, family history of diabetes, smoking and drinking status.

Model III: we adjust age, gender, height, SBP, DBP, FPG, BUN, TG, HDL-c, LDL-c, ALT, AST, family history of diabetes, smoking and drinking status.

HR, Hazard ratios; CI, confidence; Ref, reference; Cre/BW ratio, creatinine to body weight ratio.

To address potential non-linear relationships between Cre/BW ratio and prediabetes risk, we extended our analysis beyond traditional Cox proportional hazards modeling. We implemented cubic spline functions and penalized spline smoothing techniques to characterize the non-linear associations. Upon confirming non-linearity, we employed a recursive algorithm (an iterative grid-search procedure based on maximum likelihood estimation) (25) to identify the inflection point and subsequently fitted two-piecewise Cox proportional hazards models. The optimal model selection was determined through log-likelihood ratio testing (26).

We conducted stratified Cox proportional hazards analyses across key demographic and clinical parameters. Age was categorized into six groups (<30, 30-39, 40-49, 50-59, 60-69, ≥70 years), while clinical parameters were dichotomized using established thresholds (27, 28): SBP (<140, ≥140 mmHg), DBP (<90, ≥90 mmHg), and TG (<1.7, ≥1.7 mmol/L). Each stratum-specific analysis adjusted for all covariates except the stratification variable itself. Interaction effects were assessed via likelihood ratio tests by comparing models that included interaction terms with corresponding models that did not (29, 30). All subgroup analyses and interaction tests were treated as exploratory, and no adjustments for multiple comparisons (e.g., Bonferroni correction) were applied. Therefore, subgroup-specific estimates should be interpreted with caution.

We conducted comprehensive sensitivity analyses to validate our findings. The Cre/BW ratio was analyzed both as a continuous variable and categorically (quartiles) with trend testing to assess result consistency and potential non-linear relationships. Given the established associations between prediabetes risk and family history, smoking, and alcohol consumption (31–34), we performed additional analyses excluding participants with these risk factors. Due to substantial missing data (~70%) for smoking and drinking status, these variables were omitted from the multivariate model to prevent potential adjustment bias. To evaluate the robustness of our findings against unmeasured confounding, we calculated E-values (35). This methodological approach provided further insights into the reliability of our findings.

Statistical analyses were conducted using R (The R Foundation; http://www.R-project.org) and EmpowerStats (X&Y Solutions, Inc; http://www.empowerstats.com). We reported exact two-sided P-values alongside 95% confidence intervals (CIs) to evaluate the strength of the evidence, avoiding the reliance on a rigid dichotomous P-value threshold.

## Results

### Characteristics of participants

The study cohort (N = 173,476) had a mean age of 41.08 ± 12.09 years, with male predominance (53.19%). The mean Cre/BW ratio was 1.10 ± 0.22 μmol/L/kg, with participants stratified into quartiles (Q1-Q4): <0.94, 0.94-1.08, 1.09-1.24, and ≥1.24 μmol/L/kg. During median follow-up (3.00 years), 18,506 participants (10.67%) developed prediabetes. Compared with Q1, participants in Q4 had higher levels of BUN and Scr, and higher proportions of males, current smokers, and current drinkers. Conversely, the Q4 group had lower values for metabolic parameters (BMI, blood pressure, FPG, TG, TC, ALT, and AST) and a lower proportion of individuals with a family history of diabetes (**Table 2**). It should be noted that due to the exceptionally large sample size of our cohort, statistically significant P-values (<0.001) were observed across almost all baseline variables; however, these statistical differences may not necessarily reflect clinically meaningful variations.

**Table 2:** The Baseline characteristics of participants.

Cre/BW ratio quartile	Q1(<0.94)	Q2(0.94-1.08)	Q3(1.08-1.24)	Q4(≥1.24)	P-value
Participants	43369	43344	43390	43373	
Age(years)	41.2 ± 11.2	41.0 ± 11.5	40.9 ± 12.1	41.2 ± 13.5	<0.001
Height(cm)	166.5 ± 8.6	166.6 ± 8.5	166.6 ± 8.2	166.3 ± 7.9	<0.001
Weight(kg)	70.0 ± 13.4	65.0 ± 11.6	62.5 ± 10.6	58.9 ± 9.3	<0.001
BMI (kg/m^2^)	25.1 ± 3.4	23.3 ± 2.9	22.4 ± 2.7	21.2 ± 2.6	<0.001
SBP (mmHg)	119.9 ± 16.2	117.5 ± 15.7	117.0 ± 15.4	116.7 ± 15.7	<0.001
DBP (mmHg)	74.8 ± 11.1	73.5 ± 10.6	73.1 ± 10.3	72.6 ± 10.1	<0.001
FPG (mmol/L)	4.8 ± 0.5	4.8 ± 0.5	4.7 ± 0.5	4.7 ± 0.5	<0.001
TC (mmol/L)	4.7 ± 0.9	4.7 ± 0.9	4.7 ± 0.9	4.6 ± 0.9	<0.001
TG (mmol/L)	1.1 (0.8-1.7)	1.0 (0.7-1.6)	1.0 (0.7-1.5)	1.0 (0.7-1.4)	<0.001
HDL-c (mmol/L)	1.4 ± 0.3	1.4 ± 0.3	1.4 ± 0.3	1.4 ± 0.3	<0.001
LDL-c (mmol/L)	2.7 ± 0.7	2.7 ± 0.7	2.7 ± 0.7	2.7 ± 0.7	<0.001
ALT (U/L)	19.0 (13.0-31.0)	18.0 (12.5-27.8)	17.2 (12.5-25.9)	16.5 (12.2-23.3)	<0.001
AST (U/L)	22.6 (17.9-29.1)	22.0 (17.6-27.9)	21.9 (17.6-27.0)	21.7 (17.6-26.7)	<0.001
BUN (mmol/L)	4.4 ± 1.1	4.5 ± 1.1	4.7 ± 1.1	4.9 ± 1.2	<0.001
Scr (μmol/L)	58.3 ± 11.3	65.9 ± 11.9	72.1 ± 12.4	81.5 ± 13.3	<0.001
Cre/BW ratio	0.8 ± 0.1	1.0 ± 0.0	1.2 ± 0.0	1.4 ± 0.1	<0.001
Gender					<0.001
Male	16757 (38.6%)	21234 (49.0%)	24818 (57.2%)	29468 (67.9%)	
Female	26612 (61.4%)	22110 (51.0%)	18572 (42.8%)	13905 (32.1%)	
Smoking status					<0.001
Never smoker	36627 (84.5%)	35257 (81.3%)	34386 (79.2%)	33187 (76.5%)	
Ever smoker	1212 (2.8%)	1488 (3.4%)	1635 (3.8%)	1816 (4.2%)	
Current smoker	5530 (12.8%)	6599 (15.2%)	7369 (17.0%)	8370 (19.3%)	
Drinking status					<0.001
Never drinker	38358 (88.4%)	37514 (86.5%)	36831 (84.9%)	36103 (83.2%)	
Ever drinker	4436 (10.2%)	5162 (11.9%)	5805 (13.4%)	6409 (14.8%)	
Current drinker	575 (1.3%)	668 (1.5%)	754 (1.7%)	861 (2.0%)	
Family history of diabetes					<0.001
No	42266 (97.5%)	42408 (97.8%)	42555 (98.1%)	42734 (98.5%)	
Yes	1103 (2.5%)	936 (2.2%)	835 (1.9%)	639 (1.5%)	

Values are n (%), mean ± SD or medians (IQR).

BMI, body mass index; FPG, fasting plasma glucose; DBP, diastolic blood pressure; TC, total cholesterol; SBP, systolic blood pressure; TG, triglyceride; ALT, alanine aminotransferase; LDL-c, low-density lipid cholesterol; AST, aspartate aminotransferase; HDL-c, high-density lipoprotein cholesterol; BUN, blood urea nitrogen; Scr, serum creatinine; Cre/BW ratio, creatinine to body weight ratio.

Given the exceptionally large sample size of this study (N = 173,476), P-values are highly significant (<0.001) for nearly all comparisons. This phenomenon reflects high statistical power, meaning even clinically negligible differences may reach statistical significance.

**Figure 2:**
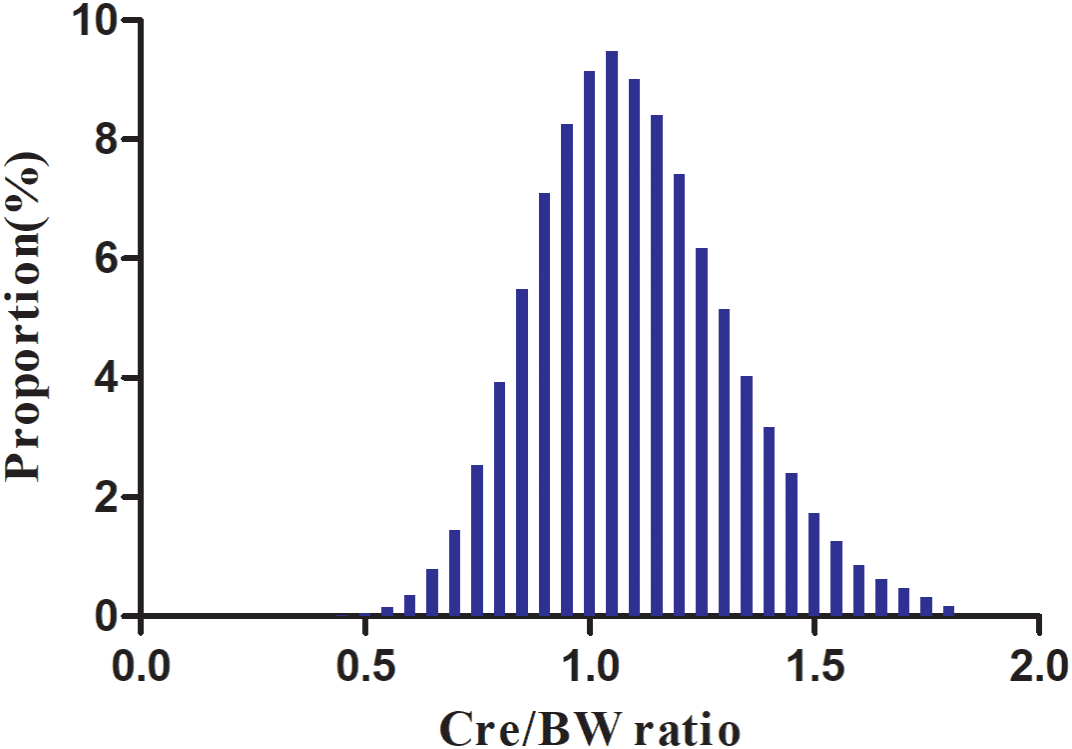
Distribution of Cre/BW ratio. Figure 2 demonstrates the statistical distribution of Cre/BW ratio levels among study participants. The ratio exhibited a normal distribution pattern, with values ranging from 0.414 to 1.810 μmol/L/kg, and a mean value of 1.098 μmol/L/kg.

**Figure 3:**
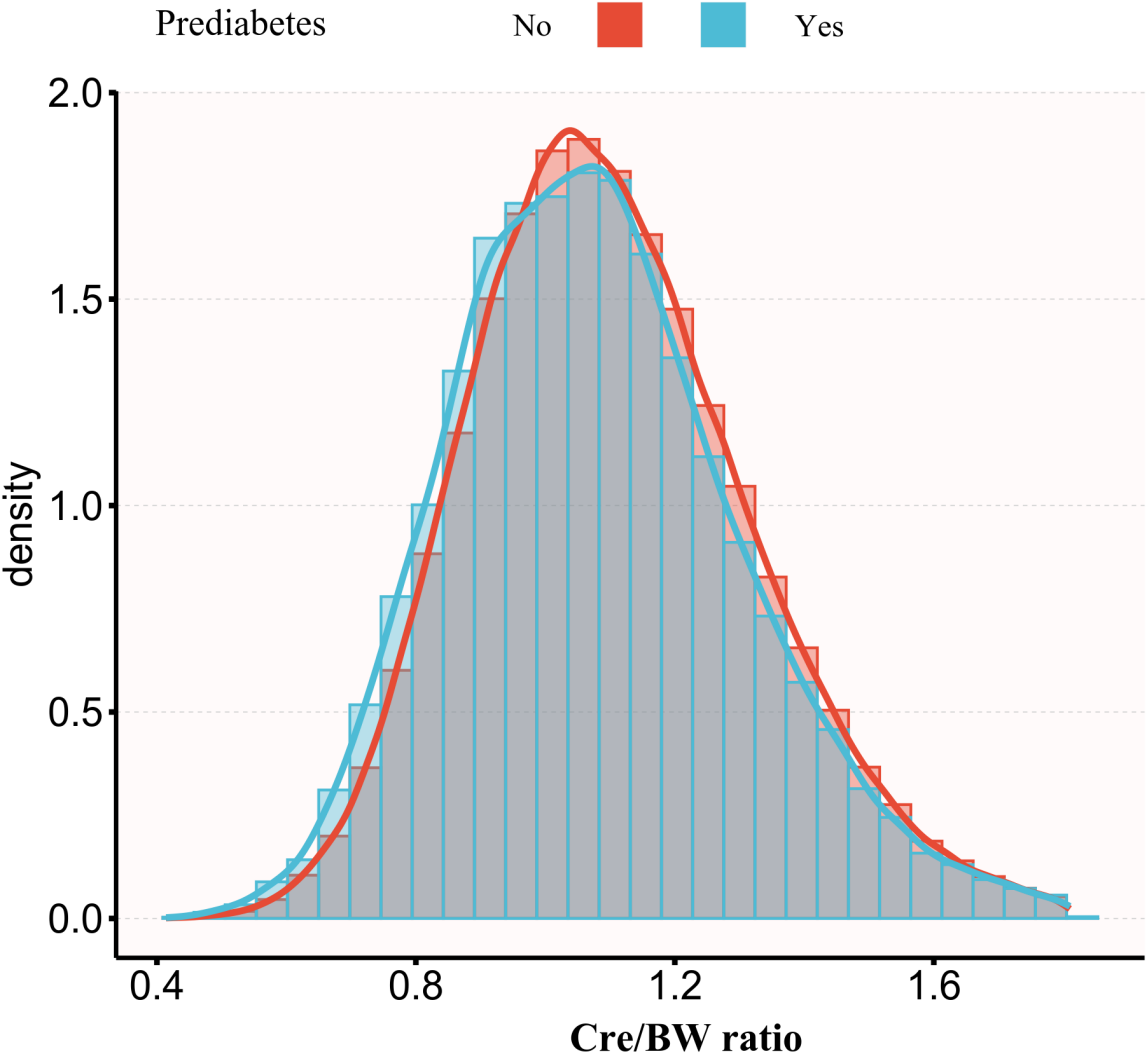
Comparison of Cre/BW ratio between groups with different prediabetes outcomes. Figure 3 illustrates the comparison of Cre/BW ratios between individuals who progressed to prediabetes and those who did not during the study period. The visualization demonstrates that participants who developed prediabetes showed lower Cre/BW ratios compared to those who maintained normal glycemic status.

The Cre/BW ratio exhibited an approximately normal distribution (range: 0.414-1.810 μmol/L/kg; mean: 1.098 μmol/L/kg; **Figure 2**). Notably, participants who progressed to prediabetes showed lower Cre/BW ratios compared to those who remained normoglycemic (**Figure 3**).

### The incidence of prediabetes

During the median 3.0-year follow-up, 18,506 participants developed prediabetes (10.67%, 95% CI: 10.52-10.81%), yielding an overall incidence rate of 3.40 per 100 person-years. A significant inverse relationship emerged between Cre/BW ratio quartiles and prediabetes risk (P<0.0001 for trend), with incidence rates declining from 3.94 (Q1) to 3.12 (Q4) per 100 person-years. The corresponding cumulative incidence decreased progressively across quartiles: 12.38% (Q1), 10.29% (Q2), 10.30% (Q3), and 9.71% (Q4) (**Table 3**).

**Table 3:** Incidence rate of prediabetes.

Cre/BW ratio	Participants(n)	Diabetes events(n)	Incidence rate (95% CI) (%)	Cumulative incidence (Per 100 person-year)
Total	173476	18506	10.67 (10.52-10.81)	3.40
Q1(<0.94)	43369	5368	12.38 (12.07-12.69)	3.94
Q2(0.94-1.08)	43344	4458	10.29 (10.00-10.57)	3.26
Q3(1.08-1.24)	43390	4468	10.30 (10.01-10.58)	3.26
Q4(≥1.24)	43373	4212	9.71 (9.43-9.99)	3.12
P for trend			<0.001	

Cre/BW ratio, creatinine to body weight ratio.

**Figure 4:**
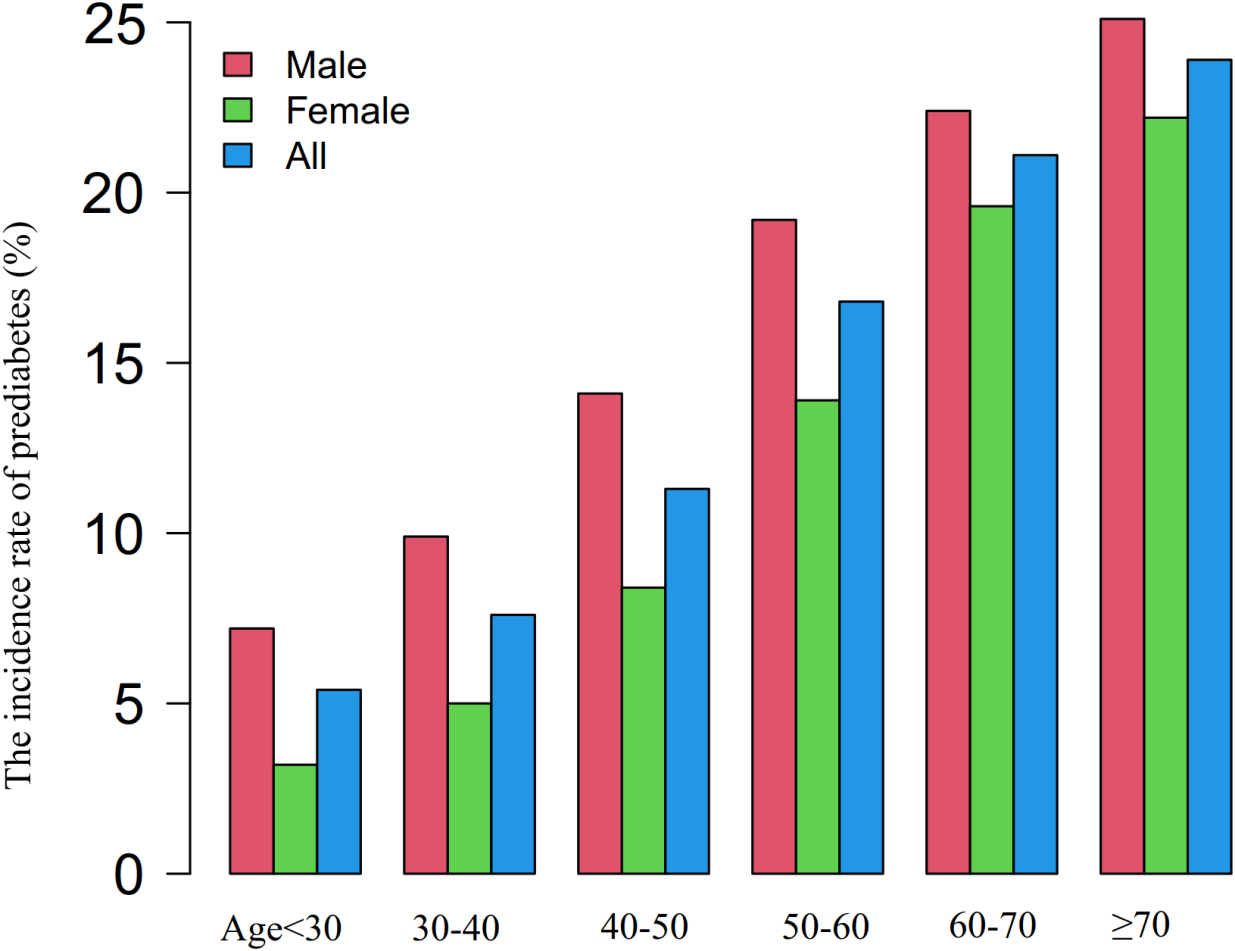
Age- and sex-stratified analysis of prediabetes incidence. Figure 4 presents a comprehensive analysis of prediabetes incidence rates across different age groups, stratified by sex. The data demonstrates that men exhibited higher incidence rates of prediabetes than women across all age groups. Moreover, both men and women showed an increase in prediabetes incidence rates with advancing age.

Age-stratified analysis revealed consistently higher prediabetes incidence in men compared to women across all decades, with both genders showing age-dependent risk elevation (**Figure 4**).

### Univariate analysis results based on Cox proportional-hazards regression

Univariate analysis identified multiple factors associated with prediabetes risk (**Table 4**). Positive associations were observed for anthropometric measures (height, weight, BMI), blood pressure (SBP, DBP), metabolic parameters (FPG, liver enzymes, lipids except HDL-c), renal function markers (BUN, Scr), and lifestyle factors (smoking, alcohol consumption). Conversely, female gender, HDL-c and Cre/BW ratio showed inverse associations. Family history of diabetes demonstrated no significant relationship with prediabetes risk.

**Table 4:** The results of univariate analysis.

Variable	Statistics	HR (95%CI)	P value
Age(years)	41.081 ± 12.088	1.033 (1.032, 1.035)	<0.00001
Gender			
Male	92277 (53.193%)	Ref.	
Female	81199 (46.807%)	0.639 (0.620, 0.658)	<0.00001
Height(cm)	166.478 ± 8.318	1.007 (1.005, 1.009)	<0.00001
Weight(kg)	64.096 ± 12.032	1.027 (1.026, 1.028)	<0.00001
BMI (kg/m^2^)	23.012 ± 3.255	1.124 (1.120, 1.129)	<0.00001
SBP (mmHg)	117.765 ± 15.795	1.025 (1.024, 1.026)	<0.00001
DBP (mmHg)	73.507 ± 10.583	1.029 (1.028, 1.030)	<0.00001
FPG (mmol/L)	4.765 ± 0.488	5.734 (5.526, 5.951)	<0.00001
TC (mmol/L)	4.673 ± 0.884	1.221 (1.203, 1.240)	<0.00001
TG (mmol/L)	1.275 ± 0.939	1.199 (1.190, 1.208)	<0.00001
HDL-c (mmol/L)	1.372 ± 0.307	0.813 (0.776, 0.851)	<0.00001
LDL-c (mmol/L)	2.692 ± 0.672	1.281 (1.256, 1.307)	<0.00001
ALT (U/L)	23.276 ± 21.573	1.003 (1.003, 1.004)	<0.00001
AST (U/L)	23.653 ± 12.051	1.006 (1.005, 1.006)	<0.00001
BUN (mmol/L)	4.603 ± 1.156	1.147 (1.134, 1.161)	<0.00001
Scr (μmol/L)	69.439 ± 14.925	1.015 (1.014, 1.016)	<0.00001
Cre/BW ratio(μmol/L/kg)	1.098 ± 0.217	0.721 (0.673, 0.772)	<0.00001
Smoking status			
Never smoker	139457 (80.390%)	Ref.	
Ever smoker	6151 (3.546%)	1.219 (1.134, 1.310)	<0.00001
Current smoker	27868 (16.064%)	1.419 (1.370, 1.470)	<0.00001
Drinking status			
Never drinker	148806 (85.779%)	Ref.	
Ever drinker	21812 (12.573%)	1.272 (1.222, 1.324)	<0.00001
Current drinker	2858 (1.647%)	1.811 (1.660, 1.977)	<0.00001
Family history of diabetes			
No	169963 (97.975%)	Ref.	
Yes	3513 (2.025%)	1.034 (0.942, 1.135)	0.47687

BMI, body mass index; FPG, fasting plasma glucose; DBP, diastolic blood pressure; TC, total cholesterol; SBP, systolic blood pressure; TG, triglyceride; ALT, alanine aminotransferase; LDL-c, low-density lipid cholesterol; AST, aspartate aminotransferase; HDL-c, high-density lipoprotein cholesterol; BUN, blood urea nitrogen; Scr, serum creatinine; Cre/BW ratio, creatinine to body weight ratio;.

HR, Hazard ratios; CI, confidence interval; Ref, reference.

For continuous variables, the reported Hazard Ratios (HRs) represent the change in risk associated with a 1-unit increase in the specified measurement scale.

**Figure 5:**
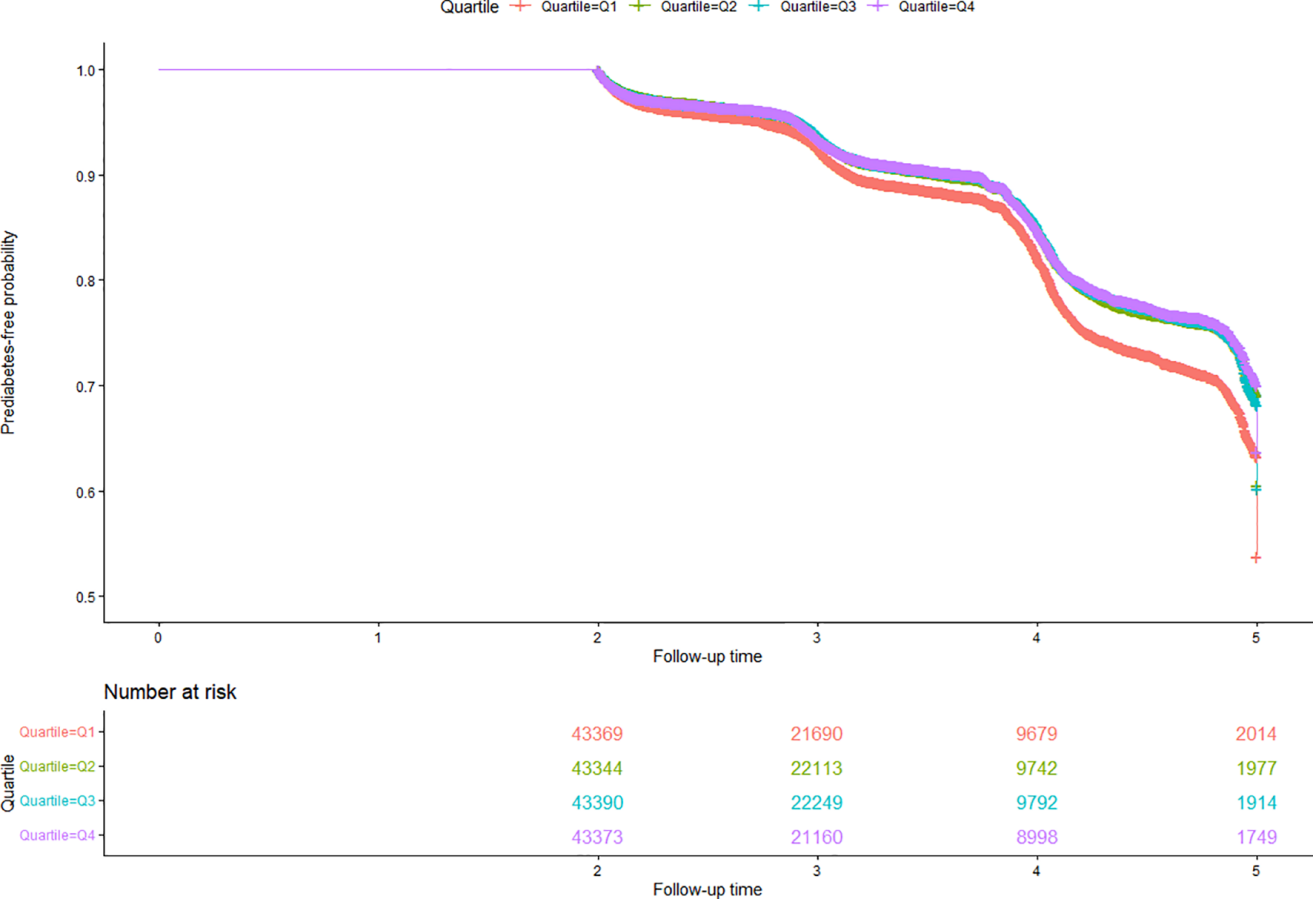
Kaplan-Meier analysis of prediabetes-free survival probability. Figure 5 presents Kaplan-Meier survival curves depicting the probability of remaining prediabetes-free, stratified by Cre/BW ratio quartiles. The analysis revealed significant differences in prediabetes-free survival probabilities between the Cre/BW ratio groups (log-rank test, P<0.0001), with higher Cre/BW ratios associated with greater prediabetes-free survival probabilities.

Kaplan-Meier analysis revealed significant differences in prediabetes-free survival across Cre/BW ratio groups (log-rank P<0.0001; **Figure 5**). Higher Cre/BW ratios were associated with improved prediabetes-free survival, suggesting a protective effect against prediabetes development.

### Results from a multivariate Cox proportional-hazards regression model

We examined the relationship between Cre/BW ratio and prediabetes risk using three sequential Cox proportional-hazards models. The unadjusted model (Model I) demonstrated that each unit (μmol/L/kg) increase in Cre/BW ratio was associated with a 27.9% reduction in prediabetes risk (HR = 0.721, 95% CI: 0.673-0.772). After adjusting for demographic variables (Model II), the protective effect strengthened, showing a 39.8% risk reduction (HR = 0.602, 95% CI: 0.559-0.648). In the fully adjusted model (Model III), which accounted for all potential confounders, the inverse association persisted, albeit attenuated, with a 13.1% decrease in prediabetes risk per unit increase in Cre/BW ratio (HR = 0.869, 95% CI: 0.806-0.973) (**Table 1**).

### Sensitivity analysis

A series of sensitivity analyses were conducted to assess the robustness of our findings. First, the Cre/BW ratio was converted into a categorical variable (quartiles), and the quartile-based Cre/BW categories were then entered into the model. **Table 1** presents the relationship between Cre/BW ratio quartiles and incident prediabetes risk. In Model 1 (crude), using the lowest quartile as the reference, the HRs (95% CIs) were 0.825 (0.793, 0.858) for quartile 2, 0.827 (0.795, 0.860) for quartile 3, and 0.822 (0.789, 0.856) for quartile 4. After adjustment for SBP, age, DBP, height, gender, smoking and drinking status, and family history of diabetes (Model II), the inverse association remained statistically significant. In Model III (fully adjusted), the corresponding HRs (95% CIs) were 0.892 (0.857, 0.929), 0.911 (0.874, 0.950), and 0.924 (0.884, 0.966), respectively, with a significant trend across quartiles (P for trend = 0.001). Notably, after categorizing Cre/BW into quartiles, the between-quartile effect sizes did not follow a completely uniform pattern, suggesting a potential non-linear association between the Cre/BW ratio and prediabetes.

The robustness of our findings was further evaluated through multiple approaches. E-value analysis (E-value=1.57) suggested minimal potential impact of unmeasured confounding on the observed associations. Supplementary analyses excluding participants with diabetes family history or lifestyle risk factors (smoking/alcohol use) consistently demonstrated the inverse relationship between Cre/BW ratio and prediabetes risk (**Table 5**).

**Table 5:** Relationship between Cre/BW ratio and prediabetes in different sensitivity analyses.

Exposure	Model I (HR,95%CI, P)	Model II (HR,95%CI, P)	Model III (HR,95%CI, P)	Model IV (HR,95%CI, P)
Cre/BW ratio (per 1 μmol/L/kg increase)	0.878 (0.814, 0.947) 0.0008	0.857 (0.785, 0.935) 0.0005	0.821 (0.756, 0.892) <0.0001	0.870 (0.807, 0.938) 0.0003
Cre/BW ratio (Quartile)				
Q1	Ref.	Ref.	Ref.	Ref.
Q2	0.896 (0.860, 0.933) <0.0001	0.890 (0.850, 0.932) <0.0001	0.889 (0.851, 0.929) <0.0001	0.892 (0.857, 0.929) <0.0001
Q3	0.915 (0.877, 0.954) <0.0001	0.891 (0.850, 0.935) <0.0001	0.882 (0.843, 0.924) <0.0001	0.911 (0.874, 0.950) <0.0001
Q4	0.929 (0.888, 0.972) 0.0013	0.924 (0.878, 0.973) 0.0027	0.900 (0.857, 0.945) <0.0001	0.924 (0.884, 0.966) 0.0005
P for trend	0.0029	0.0013	<0.0001	0.0012

Model I was sensitivity analysis in participants without family history of diabetes (N = 169,963). We adjusted age, gender, height, SBP, DBP, FPG, BUN, TG, HDL-c, LDL-c, ALT, AST, smoking and drinking status.

Model II was a sensitivity analysis performed on never smoker participants (N = 139,457). We adjusted age, gender, height, SBP, DBP, FPG, BUN, TG, HDL-c, LDL-c, ALT, AST, family history of diabetes, and drinking status.

Model III was a sensitivity analysis performed on never drinker participants (N = 148,806). We adjusted age, gender, height, SBP, DBP, FPG, BUN, TG, HDL-c, LDL-c, ALT, AST, family history of diabetes, and smoking status.

Model IV was sensitivity analysis in participants without adjusting smoking and drinking status (N = 173,476). We adjusted age, gender, height, SBP, DBP, FPG, BUN, TG, HDL-c, LDL-c, ALT, AST, family history of diabetes.

HR, Hazard ratios; CI, confidence; Ref, reference; Cre/BW ratio, creatinine to body weight ratio.

Given the high proportion of missing data for smoking and drinking status (approximately 70%), we performed additional analyses that excluded these covariates from the multivariable model. The association was essentially unchanged (HR = 0.870, 95% CI: 0.807–0.938), further confirming the robustness of the primary results (**Table 5**).

### Cox proportional hazards regression incorporating cubic spline functions to model non-linearity

**Figure 6:**
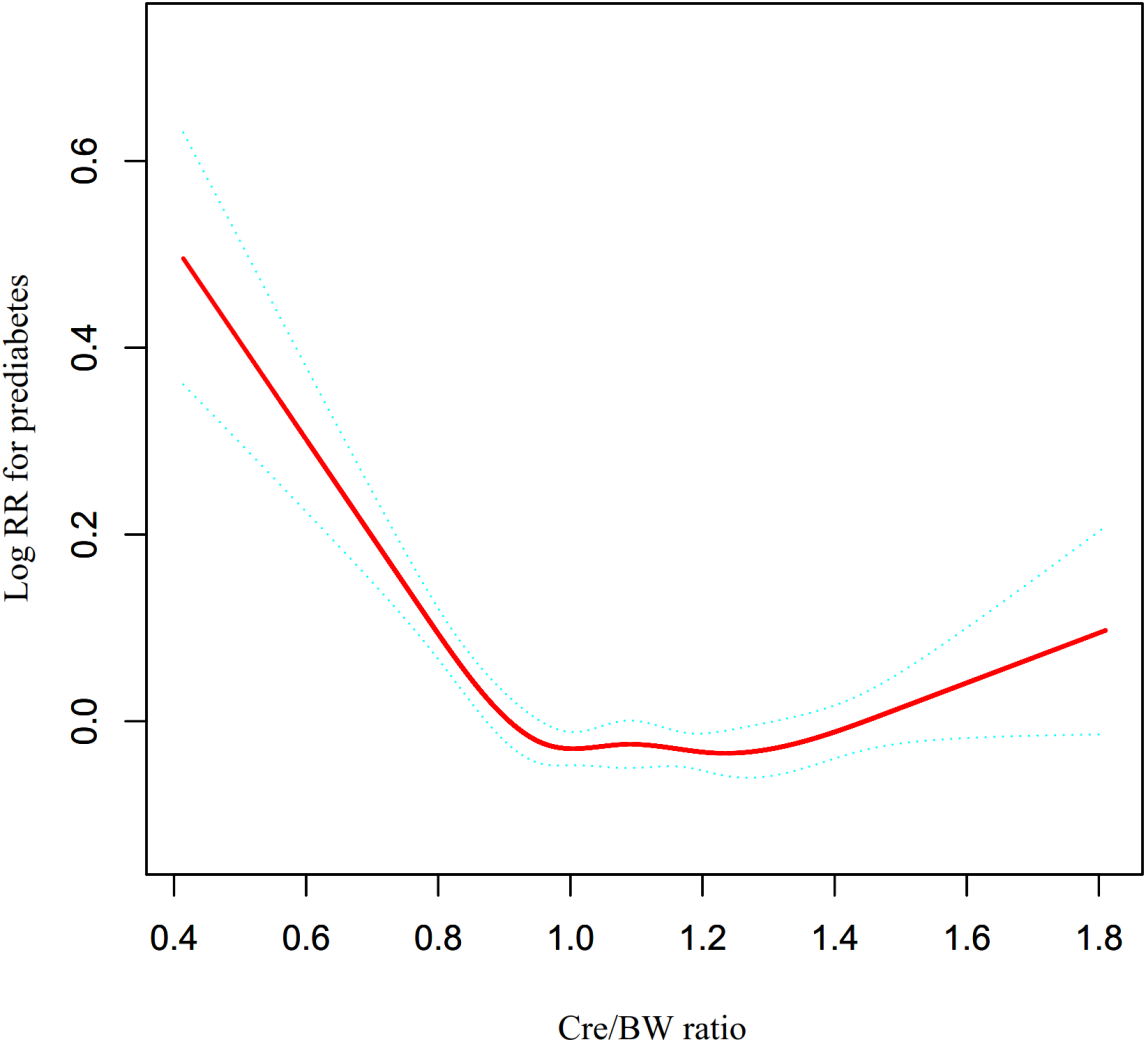
Non-linear association between Cre/BW ratio and prediabetes risk. Figure 6 illustrates the complex relationship between Cre/BW ratio and the risk of prediabetes, analyzed using Cox proportional hazards regression with cubic spline functions. The analysis revealed a non-linear association characterized by an inflection point at 0.96(95% CI 0.90-1.01) μmol/L/kg, below which the association was notably stronger (HR = 0.407, 95%CI: 0.328-0.506) compared to above the inflection point (HR = 1.092, 95%CI: 0.992-1.202).

Cox proportional hazards models incorporating cubic spline functions indicated a non-linear association between the Cre/BW ratio and prediabetes risk (**Figure 6**). A subsequent two-piecewise Cox regression further demonstrated a significant threshold effect (P < 0.05; **Table 6**). Using a recursive algorithm, an inflection point was estimated at 0.96 (95% CI: 0.90–1.01) μmol/L/kg, separating two distinct patterns: below this value, each unit increase in the Cre/BW ratio was linked to a marked reduction in risk (HR = 0.407, 95% CI: 0.328–0.506), whereas above the inflection point the association was not statistically significant (HR = 1.092, 95% CI: 0.992–1.202).

**Table 6:** The result of the two-piecewise Cox regression model.

Incident diabetes	Model I (HR,95%CI, P)
Fitting model by standard Cox regression	0.869 (0.806, 0.937) 0.0003
Fitting model by two-piecewise Cox regression	
Inflection point of the Cre/BW ratio	0.96(95% CI 0.90-1.01)
≤Inflection point	0.407 (0.328, 0.506) <0.0001
>Inflection point	1.092 (0.992, 1.202) 0.0739
P for log-likelihood ratio test	<0.001

We adjusted age, gender, height, SBP, DBP, FPG, BUN, TG, HDL-c, LDL-c, ALT, AST, family history of diabetes, smoking and drinking status.

HR, Hazard ratios; CI, confidence; Ref, reference; Cre/BW ratio, creatinine to body weight ratio.

### The results of subgroup analyses

Comprehensive evaluation of effect modification across prespecified and exploratory subgroups revealed significant interactions with age, gender, and SBP, while no significant interactions were observed for TG or DBP (**Table 7**).

**Table 7:** Effect size of the Cre/BW ratio on incident diabetes in prespecified and exploratory subgroups.

Characteristic	No of participants	HR (95%CI)	P value	P for interaction
Age(years)				<0.0001
20 to <30	25112	0.953 (0.731, 1.244)	0.7259	
30 to <40	71858	0.614 (0.532, 0.708)	<0.0001	
40 to <50	37168	0.970 (0.822, 1.145)	0.7226	
50 to <60	22256	1.008 (0.848, 1.198)	0.9322	
60 to <70	12427	1.140 (0.939, 1.384)	0.1853	
≥70	4655	1.287 (0.982, 1.686)	0.0673	
Gender				0.0003
Male	92277	0.968 (0.882, 1.063)	0.4959	
Female	81199	0.726 (0.640, 0.824)	<0.0001	
TG(mmol/L)				0.0767
<1.7	137582	0.849 (0.777, 0.927)	0.0003	
≥1.7	35894	0.975 (0.855, 1.111)	0.7048	
SBP (mmHg)				0.0467
<140	159014	0.816 (0.752, 0.886)	<0.0001	
≥140	14462	0.970 (0.830, 1.134)	0.7046	
DBP (mmHg)				0.3021
<90	161355	0.853 (0.788, 0.924)	<0.0001	
≥90	12121	0.949 (0.784, 1.148)	0.5885	

1: Above model adjusted for age, gender, height, SBP, DBP, FPG, BUN, TG, HDL-c, LDL-c, ALT, AST, family history of diabetes, smoking and drinking status.

2: In each case, the model is not adjusted for the stratification variable.

The protective association between Cre/BW ratio and prediabetes risk was most pronounced among adults aged 30–40 years (HR = 0.614, 95% CI: 0.532-0.708), females (HR = 0.726, 95% CI: 0.640-0.824), and individuals with SBP <140 mmHg (HR = 0.816, 95% CI: 0.752-0.886). This association was attenuated or absent in other age groups (HRs ranging from 0.953 to 1.287), males (HR = 0.968, 95% CI: 0.882-1.063), and those with SBP ≥140 mmHg (HR = 0.970, 95% CI: 0.830-1.134).

## Discussion

In this large retrospective cohort of 173,476 Chinese adults, a lower Cre/BW ratio was associated with a higher risk of prediabetes. The association remained statistically significant after adjustment for multiple potential confounders. Moreover, the Cre/BW ratio showed a non-linear relationship with prediabetes risk, with an estimated inflection point of 0.96 (95% CI: 0.90–1.01) μmol/L/kg. Furthermore, this association was more pronounced among individuals aged 30–40 years, females, and those with SBP less than 140 mmHg.

During a median follow-up of 3.0 years, the cumulative incidence of prediabetes in our cohort was 10.67%, yielding a incidence rate of 3.40 per 100 person-years. This rate is somewhat lower than that reported in other Chinese cohort studies (4). The discrepancy may be attributable to differences in study design and participant characteristics, such as the younger mean age of our population (41.08 years), exclusion of individuals with baseline FPG ≥ 5.6 mmol/L, and the shorter follow-up duration (7).

Our findings regarding the inverse association between the Cre/BW ratio and prediabetes risk align with several previous studies examining the relationship between muscle mass and glucose metabolism disorders. Hashimoto et al. reported that a lower Cre/BW ratio was associated with an increased risk of type 2 diabetes in a Japanese population (13). Similarly, Srikanthan et al. demonstrated that relative muscle mass was inversely associated with insulin resistance and prediabetes using NHANES data (8). Moon et al. further showed that low muscle mass was associated with an increased risk of diabetes development in Korean adults (36). Our study extends these findings by specifically focusing on prediabetes in a large Chinese population and identifying important effect modifications by age, gender, and blood pressure. Concurrently, sensitivity analyses showed that the association remained consistent in never-smokers/never-drinkers and in participants without a family history of diabetes. In addition, when smoking and drinking status were excluded from the multivariable regression model, the inverse association between the Cre/BW ratio and prediabetes risk persisted. Overall, these analyses further supported the robustness of the observed relationship between the Cre/BW ratio and prediabetes. This is consistent with recent literature emphasizing the critical crosstalk between skeletal muscle and systemic glucose homeostasis, where muscle acts not only as a primary site for glucose disposal but also as an endocrine organ (37). Furthermore, recent findings highlight that preserving muscle mass, as reflected by higher Cre/BW, serves as a protective factor against the progression from normoglycemia or prediabetes to overt diabetes (38).

Several potential mechanisms might explain the observed association between low Cre/BW ratio and increased prediabetes risk. Skeletal muscle, the body’s largest insulin-sensitive tissue, is essential for maintaining glucose homeostasis (10, 39). Lower muscle mass, as indicated by a lower Cre/BW ratio, might lead to reduced glucose uptake and disposal capacity, contributing to insulin resistance and eventual prediabetes (11, 40). Additionally, skeletal muscle serves as an endocrine organ, secreting myokines such as interleukin-6, irisin, and myonectin that influence whole-body metabolism and insulin sensitivity (41, 42). Furthermore, a lower muscle mass might reflect a more sedentary lifestyle or poor nutritional status, both of which are established risk factors for prediabetes (43, 44). However, it must be explicitly stated that these mechanistic parameters-including direct measurements of muscle mass, insulin resistance, and specific myokine levels-were not evaluated in our current study cohort, and these explanations remain theoretical. In addition, it is important to interpret the Cre/BW ratio with caution due to the mathematical coupling between the numerator (creatinine) and the denominator (body weight). Since body weight is a strong determinant of insulin resistance, the protective association observed in our study may reflect the combined effects of higher muscle mass and lower adiposity, rather than the physiological benefits of muscle tissue alone. Therefore, the Cre/BW ratio should be viewed as a composite surrogate marker of body composition.

Exploratory threshold effect analysis suggested a potential inflection point at 0.96(95% CI 0.90-1.01) μmol/L/kg, suggests that the protective association of muscle mass might reach a plateau. Below this inflection point, increases in the Cre/BW ratio are associated with substantial reductions in prediabetes risk (HR = 0.407, 95% CI: 0.328-0.506), while above this level, the relationship becomes non-significant (HR = 1.092, 95% CI: 0.992-1.202). This pattern might reflect the complex interplay between muscle mass, body composition, renal function, and metabolic health (45–47). Similar non-linear relationships have been observed in studies examining the association between muscle mass and other metabolic outcomes (48). However, this inflection point was derived from a data-driven approach in the current cohort and should be interpreted as exploratory. We caution against viewing this specific value as a definitive clinical cutoff without external validation. The inflection point likely represents a saturation effect where the protective benefit of muscle mass plateaus, rather than a sharp diagnostic boundary.

Our exploratory subgroup analysis suggested stronger association observed in females compared to males might be explained by several factors. Women generally have lower muscle mass than men, and the impact of muscle loss on metabolic health might be more pronounced in this population (49). Additionally, hormonal differences and varying patterns of fat distribution between genders could modify the relationship between muscle mass and glucose metabolism (50). The age-specific effect modification, with the strongest association in the 30–40 year age group, might reflect the critical period when lifestyle interventions could be most effective in preventing metabolic disorders (51).

Our findings may have several potential clinical implications. First, the Cre/BW ratio has the potential to serve as a simple, cost-effective supplementary indicator for identifying individuals at high risk of prediabetes, particularly in resource-limited settings where more sophisticated measures of muscle mass are not readily available (52). Second, the identification of high-risk subgroups (ages 30-40, females, and those with normal blood pressure) may assist in informing targeted preventive strategies. Third, our findings suggest that maintaining adequate muscle mass might be a relevant factor in prediabetes prevention, highlighting the need for future prospective trials to evaluate whether incorporating resistance training could be beneficial in diabetes prevention programs (53, 54).

Our study has several strengths. First, it is one of the largest cohort studies examining the relationship between the Cre/BW ratio and prediabetes risk. Second, we employed robust statistical methods, including multiple sensitivity analyses and careful adjustment for confounders. Third, in this study, missing data were handled using multiple imputations. Using multiple imputations could increase statistical power and reduce any potential bias brought on by the absence of covariate information.

However, several limitations should be acknowledged. First, residual confounding remains a concern due to the retrospective design. We lacked data on key lifestyle factors, including physical activity (specifically resistance training), dietary habits (especially protein intake), and socioeconomic status. Physical activity is a strong determinant of both muscle mass and insulin sensitivity, while dietary protein intake directly influences serum creatinine levels. Given the modest E-value observed in our analysis, we cannot rule out the possibility that unmeasured confounding—such as a healthy user bias where individuals with higher muscle mass also engage in healthier behaviors—may explain part of the observed association (55). Therefore, our findings should be interpreted as observational associations rather than causal effects. Second, we lacked information on physical activity and dietary habits, which could influence both muscle mass and prediabetes risk (56). The Cre/BW ratio, while practical, is an indirect measure of muscle mass and might be influenced by factors such as kidney function and protein intake (57–59). Third, the median follow-up period was approximately 3 years, which limits our ability to observe the long-term natural history of disease development. Consequently, our findings reflect the short-term risk of incident prediabetes. Furthermore, as shown in our stratified analysis (**Table 7**), the association was predominantly observed in adults aged 30–40 years, indicating that the risk is age-conditional. Future studies with longer follow-up are needed to determine if these associations persist into later life stages. Fourth, our findings from a Chinese population might not be fully generalizable to other ethnic groups due to potential differences in body composition and lifestyle factors (60). Fifth, we acknowledge the potential for mathematical coupling inherent in the Cre/BW ratio. Because body weight serves as the denominator, an elevated ratio could result from either high serum creatinine (reflecting muscle mass) or low body weight. Given that lower body weight is generally protective against prediabetes, the observed inverse association might be partially driven by the denominator effect. While we adjusted for height and lipid profiles to control for body size and metabolic status, we did not adjust for BMI to avoid multicollinearity. Consequently, our findings should be interpreted as an association with a composite body composition index rather than a direct causal effect of skeletal muscle mass. Sixth, we conducted multiple subgroup analyses to explore potential effect modifiers. Since we did not adjust for multiplicity, there is an inherent risk of Type I error (false positives). Consequently, these subgroup findings should be considered exploratory and hypothesis-generating, requiring validation in independent cohorts before guiding targeted interventions. Furthermore, the Cre/BW ratio was calculated based on a single measurement at baseline. Due to the inherent short-term biological variability in serum creatinine and body weight, reliance on a single time point may introduce regression dilution bias. This type of non-differential measurement error typically attenuates the exposure-outcome association towards the null, suggesting that the true association between the Cre/BW ratio and the risk of prediabetes might be stronger than the estimates observed in our study. Consequently, our findings should be interpreted with caution, acknowledging that the effect sizes may be underestimated. Finally, excluding participants with baseline FPG≥5.6 mmol/L was necessary to determine incident prediabetes. We acknowledge that this restriction conditions the analysis on a variable potentially influenced by the exposure, which could theoretically introduce collider bias. However, we adjusted for baseline FPG in our multivariable models to mitigate this potential bias, and the results remained consistent. Nevertheless, the observed associations should be interpreted with caution regarding causality.

## Conclusion

In conclusion, our observational study demonstrates that a lower Cre/BW ratio is significantly associated with an increased risk of prediabetes in Chinese adults, particularly among individuals aged 30–40 years, females, and those with normal blood pressure, exhibiting a non-linear pattern. Given the retrospective nature of this analysis, our findings are inherently hypothesis-generating and cannot establish causality. Further studies are needed to determine if a biologically relevant threshold exists for clinical utility. These findings suggest that the Cre/BW ratio might serve as a useful tool for prediabetes risk stratification and highlight the importance of maintaining adequate muscle mass for metabolic health. Future studies should focus on: (1) validating these associations in different populations and prospective cohorts; (2) exploring the underlying biological mechanisms linking muscle-related biomarkers to glucose dysregulation; and (3) investigating the potential benefits of muscle-strengthening interventions in prediabetes prevention through well-designed randomized controlled trials.

## Data Availability

The datasets presented in this study can be found in online repositories. The names of the repository/repositories and accession number(s) can be found in the article/**Supplementary Material**.
