# Supplementary material for: Association between creatinine-to-body weight ratio and incident prediabetes in Chinese adults: a large-scale retrospective cohort study
Source: Front Endocrinol (Lausanne). 2026 Apr 2;17:1713333. doi: 10.3389/fendo.2026.1713333 (PMC13083015; doi:10.3389/fendo.2026.1713333)
Supplement: Supplementary file 1 [file Table1.docx]

**Association Between Creatinine-to-Body Weight Ratio and Incident Prediabetes in Chinese Adults: A Large-Scale Retrospective Cohort Study**

**Running title：Cre/BW and risk of prediabetes**

**Wushan Pan^1^****^#^, Yuheng Liao^2,3#^, Yong Han****^4,5#^, Changchun Cao^6,7*^, Haofei Hu ^2,3*^**

^1^Department of Nephrology, Kaifeng Central Hospital, Kaifeng 475000, Henan Province, China

^2^Department of Nephrology, Shenzhen Second People’s Hospital, Shenzhen 518000, Guangdong Province, China

^3^Department of Nephrology, The First Affiliated Hospital of Shenzhen University, Shenzhen 518000, Guangdong Province, China

^4^Department of Emergency, Shenzhen Second People’s Hospital, Shenzhen 518000, Guangdong Province, China

^5^Department of Emergency, The First Affiliated Hospital of Shenzhen University, Shenzhen 518000, Guangdong Province, China

^6^Department of Rehabilitation, The First Affiliated Hospital of Shenzhen University, Shenzhen 518000, Guangdong Province, China

^7^Department of Rehabilitation, Shenzhen Second People’s Hospital, Shenzhen 518000, Guangdong Province, China

**^#^These authors contributed equally: Wushan Pan, Yuheng Liao and Yong Han**

***Corresponding author**

Changchun Cao

Department of Rehabilitation,

Shenzhen Second People’s Hospital,

No.3002 Sungang Road, Futian District,

Shenzhen 518000,

Guangdong Province,

China

Tel:+86-755-83366388

E-mail: caochangchun1015@163.com

***Corresponding author**

**Haofei Hu**

Department of Nephrology,

Shenzhen Second People’s Hospital,

No.3002 Sungang Road, Futian District,

Shenzhen 518000,

Guangdong Province,

China

Tel:+86-755-83366388

E-mail: huhaofei0319@126.com

**Table S1. Collinearity diagnostics steps.**

| Variable | VIF  Step 1 | Step 2 |
| --- | --- | --- |
|  |  |  |
| Gender | 2.7 | 2.7 |
| Age(years) | 1.4 | 1.3 |
| Smoking status | 1.3 | 1.3 |
| Drinking status | 1.1 | 1.1 |
| ALT(U/L) | 3.1 | 3.1 |
| AST(U/L) | 2.8 | 2.8 |
| Family history of diabetes | 1.0 | 1.0 |
| BUN(mmol/L) | 1.1 | 1.1 |
| FPG(mmol/L) | 1.1 | 1.0 |
| TC(mmol/L) | 7.7 | NA |
| TG(mmol/L) | 1.7 | 1.3 |
| HDL-c(mmol/L) | 1.5 | 1.2 |
| LDL-c(mmol/L) | 6.3 | 1.1 |
| Height(cm) | 2.1 | 2.1 |
| SBP(mmHg) | 2.1 | 2.1 |
| DBP(mmHg) | 2.0 | 2.0 |

SBP, Systolic blood pressure; DBP, Diastolic blood pressure; ALT, Alanine aminotransferase; AST, Aspartate aminotransferase; TC, Total cholesterol; TG, Triglyceride; HDL-c, High-density lipoprotein cholesterol; LDL-c, Low-density lipid cholesterol; BUN, Serum urea nitrogen; FPG, Fasting plasma glucose;

Abbreviation: VIF: variance inflation factor; VIF = 1/(1-R^2^).

Note: The variables with VIF>5 will be regarded as collinear variables and cannot be included in the multiple regression model.
